# Decoupled Glucose and Lipid Metabolic Recovery after Viral Clearance in Direct-Acting Antiviral-Treated HCV Patients: A 3-Year Prospective Cohort Study

**DOI:** 10.3390/cells10112934

**Published:** 2021-10-28

**Authors:** Heng Lee, Rong-Nan Chien, Li-Heng Pao, Chia-Jung Kuo, Po-Han Huang, Ming-Ling Chang

**Affiliations:** 1 Department of Medicine, College of Medicine, Chang Gung University, Taoyuan 333323, Taiwan; hlee8@vghtpe.gov.tw (H.L.); ronald@adm.cgmh.org.tw (R.-N.C.); m7011@cgmh.org.tw (C.-J.K.); Cksai2005@yahoo.com.tw (P.-H.H.); 2 Department of Medicine, Taipei Veterans General Hospital, Taipei 112201, Taiwan; 3 Division of Hepatology, Department of Gastroenterology and Hepatology, Chang Gung Memorial Hospital, Taoyuan 333423, Taiwan; 4 Graduate Institute of Health Industry Technology, Chang Gung University of Science and Technology, Taoyuan 33303, Taiwan; paolhaa@gmail.com; 5 Research Center for Food and Cosmetic Safety and Research Center for Chinese Herbal Medicine, College of Human Ecology, Chang Gung University of Science and Technology, Taoyuan 33303, Taiwan

**Keywords:** HCV, metabolic alteration, lipid, HOMA-IR, DAA

## Abstract

Background/Aim: The recovery pattern of hepatitis C virus (HCV)-associated metabolic alteration after sustained virological response (SVR) following direct-acting antivirals (DAAs) remains elusive. Methods: A prospective cohort study of chronic HCV-infected (CHC) patients (n = 415) receiving DAAs (n = 365) was conducted. Metabolic profiles were examined in SVR patients (n = 360) every 3–6 months after therapy and compared with those of sex- and age-matched controls (n = 470). Results: At baseline, of 415, 168 (40.5%) had insulin resistance (IR). The following were associated: levels of high-density lipoprotein cholesterol (HDL-C), triglycerides (TGs), HCV RNA, fibrosis-4 score, and interferon-λ3-rs12979860 genotype with total cholesterol (TC) levels; and TG levels and BMI with HOMA-IR. Over a 3-year follow-up, in SVR patients, BMI and TC levels and TG/HDL-C ratios increased from baseline, while HOMA-IR trended downward by 72 weeks after therapy and then increased. The increased HDL-C levels began to decrease after 72 weeks after therapy. TC and HOMA-IR were negatively associated with each other until 24 weeks after therapy. Earlier increases in BMI and decreases in HOMA-IR were noted in SVR patients with than in those without baseline IR. Compared with controls, in the subgroup without baseline IR, SVR patients had increased BMI and HOMA-IR levels. Metabolic profiles were similar between SVR patients and controls in the subgroup with baseline IR. Conclusions: In SVR patients treated with DAAs, the recovery of altered lipid and glucose metabolism was not coupled until 72-week post-therapy, when HOMA-IR reached its nadir. SVR patients with baseline IR recovered from HCV-associated metabolic alterations earlier than those without baseline IR.

## 1. Introduction

Hepatitis C virus (HCV), classified into eight genotypes [1], is a human pathogen that causes acute and chronic liver disease, chronically infecting an estimated 71.1 million individuals worldwide [2]. In addition to hepatic complications that include steatosis, liver cirrhosis, and hepatocellular carcinoma (HCC), HCV infection induces several extrahepatic complications, such as hypolipidemia, insulin resistance (IR), diabetes, and cardiovascular events [3,4]. Accordingly, HCV infection is now considered to cause metabolic alterations instead of being simply a viral infection.

The reversal of HCV-associated metabolic alterations after sustained virological response (SVR) in interferon-based studies has been well demonstrated [3]. However, HCV-associated diabetes and some cardiovascular events cannot be reversed, even after viral clearance [3]. HCV-associated HCC risk is also not completely prevented, especially among patients with baseline diabetes and cirrhosis [5]. Specifically, hyperactive lipogenesis [6] substantially contributes to the development of malignancies [7] and cardiovascular events [8]. Diabetes and IR are associated with various types of cancer and are risk factors for cancer-specific and all-cause mortality in postmenopausal women [9]. Thus, the recovery patterns of HCV-associated metabolic alterations including hypolipidemia and IR, namely, degrees of reversal of hypolipidemia and IR, might serve as targets for the treatment of currently irreversible complications of HCV [3]. However, these patterns might be biased by interferon-based therapy, as interferon therapy has been associated with increases in lipid levels [10] and with immune modulation in patients [11]. With the advent of direct-acting antivirals (DAAs), which target specific proteins of HCV during its life cycle [12], anti-HCV treatment has resulted in a high cure rate with a short course of treatment in patients with chronic HCV infection (CHC), and the recovery pattern of HCV-associated metabolic alterations may not be masked by any effect of interferons. Although several studies have shown the reversal of hypocholesterolemia in CHC patients after SVR following DAA therapy [13,14], there are inconsistent data regarding other lipids, including triglycerides (TGs) and high-density lipoprotein cholesterol (HDL-C) [13,14,15,16,17,18,19]. Moreover, there is conflicting evidence on whether IR improves after SVR following DAA therapy [13,20,21,22,23,24]. The changes in homeostatic model assessment for insulin resistance (HOMA-IR) values between SVR patients with and without baseline IR following interferon-based therapy were different [25]. In addition, the follow-up durations were quite variable, ranging from 12 to 48 weeks, in studies regarding metabolic alterations in CHC patients who had undergone DAA therapy [13,20,21,22,23,24]. The heterogeneity of host baseline metabolic profiles, particularly IR status, and the variability of post-SVR follow-up durations might account for the inconsistent recovery of metabolic alterations among CHC patients in previous studies.

Accordingly, we sought to elucidate the recovery pattern of HCV-associated metabolic alterations by conducting a prospective study to analyze the metabolic profiles of CHC patients before and after DAA-based therapy. Patients were followed up to 3 years. Subgroup analyses were performed according to patients’ baseline IR. In parallel, metabolic profiles of sex- and age-matched controls were used to verify the complete reversal of metabolic alterations after viral clearance.

## 2. Materials and Methods

### 2.1. Patients

The study group comprised subjects aged >18 years who had CHC, defined as detectable HCV RNA by PCR for >24 weeks. The control group comprised subjects aged >18 years who had no HCV infection. Controls were matched with the members of the study group according to sex and age. Subjects with human immunodeficiency virus or hepatitis B virus infection, hemochromatosis, primary biliary cholangitis, primary sclerosing cholangitis, autoimmune hepatitis or malignancy, and recipients of solid organ transplants were excluded, as were those on lipid-lowering or glucose-lowering medications.

### 2.2. Study Design

**Figure 1:**
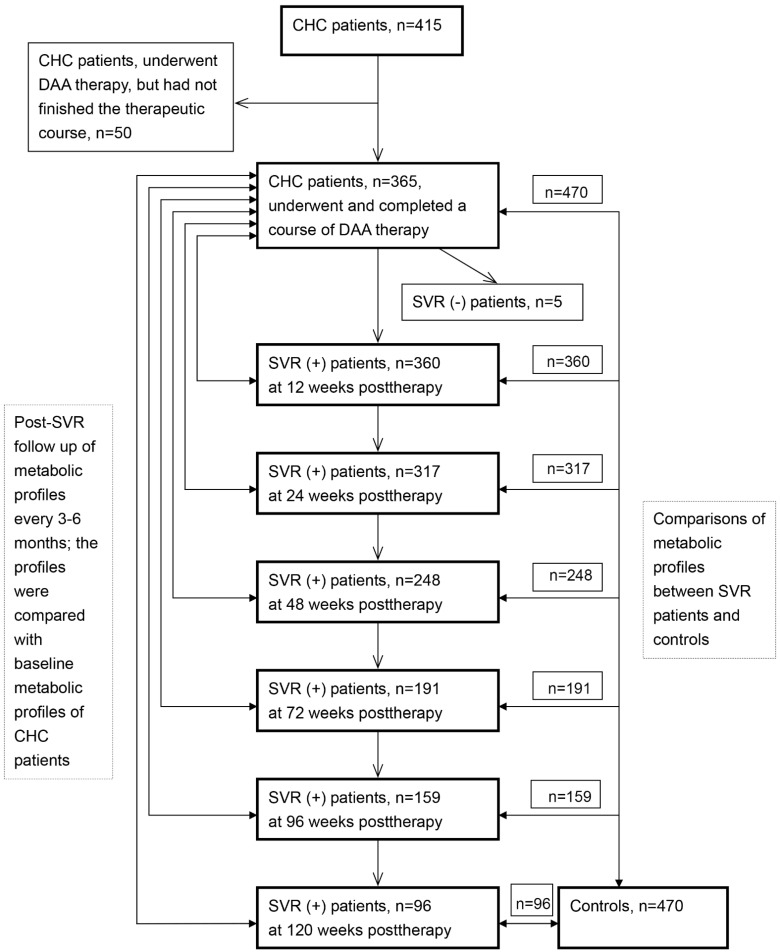
Schematic flow chart of the enrolled patients. CHC: chronic hepatitis C virus infection; DAA: direct-acting antiviral; SVR: sustained virological response.

A schematic flow chart of all the enrolled subjects is shown in Figure 1. In total, 415 CHC patients and 470 control patients were consecutively recruited at a tertiary referral center between January 2017 and April 2020. The control patients were enrolled from those who underwent health checkups and had been confirmed without HCV infection. Of 415 CHC patients, 365 had completed a course of anti-HCV therapy with various DAA combinations (Supplementary Materials, Table S1) and had been followed for >12 weeks. Several baseline factors, including sex, age, body mass index (BMI), HCV genotype, hepatic cirrhosis, HCV RNA, alanine aminotransferase (ALT), fibrosis-4 (FIB-4) score, HOMA-IR, total cholesterol (TC), TG, HDL-C, TG/HDL-C ratio, and interferon-λ3 (IFNL3)-rs12979860 single-nucleotide polymorphisms genotype, were surveyed and recorded in all CHC patients at enrollment. Biochemical tests were performed using routine automated techniques in the clinical pathology laboratories of the hospital. BMI [weight/(height)^2^ kg/m^2^] categories defined normal weight (BMI < 24), overweight (24 ≤ BMI < 27), and obesity (BMI ≥ 27), according to recommendations from the Bureau of Health Promotion of Taiwan [26]. HOMA-IR ([fasting insulin (µU/mL) × fasting glucose (mmol/L)]/22.5)) is an indicator used to survey insulin resistance (IR). Baseline HOMA-IR >2.5 was defined as IR, and the higher HOMR-IR levels, the worse IR [25]. Diabetes was defined as hemoglobin A1c ≥ 6.5% or fasting plasma glucose level ≥126 mg/dL [27]. FIB-4 identifies fibrosis stages, long-time prognosis in chronic liver disease, and short-time outcomes in acute liver injury. FIB-4 was accurate in predicting the absence or presence of advanced fibrosis with cut-offs of 1.45 and 3.25 for viral hepatitis C, and 1.30 (<65 years), 2.0 (≥65 years), and 2.67 for non-alcoholic fatty liver disease [28]. The TG/HDL-C ratio is a predictor of coronary disease development [29] and an indicator of IR [30,31]. An SVR was defined as an undetectable level of HCV RNA 12 weeks after the completion of therapy. For the CHC patients who had completed a course of DAA-based therapy and achieved an SVR, the aforementioned variables were measured at 3 months and again every 6 months after the completion of anti-HCV therapy.

### 2.3. Statistics

All statistical analyses were performed using the Statistical Package for the Social Sciences (SPSS version 21, SPSS Inc., Chicago, IL, USA) or MedCalc (MedCalc ver. 12.4, MedCalc Software Corp., Acacialaan, Ostend, Belgium) software. Continuous variables are summarized as the means ± standard deviations, and categorical variables are summarized as numbers and percentages (%). For comparisons between groups, continuous variables were analyzed using Student’s t-tests, whereas categorical variables were analyzed using the chi-squared test or Fisher’s exact test as appropriate. Multivariate stepwise linear regression models were used to assess the relationships between various dependent and independent variables. The variables found to be significantly associated with the dependent variables in univariate analyses were included in multivariate regression models (all *p* values < 0.05). Longitudinal alterations in metabolic profiles (baseline, 12 weeks, and every 24 weeks after therapy) within the same SVR patients were analyzed and compared using analysis of variance, employing repeated-measures general linear models. Paired t-tests or nonparametric methods (Wilcoxon test) were performed for the same variable measured in the same subjects at >2 time points if Mauchly’s sphericity test yielded *p* values < 0.05 during repeated measures. Statistical significance was defined at the 5% level based on two-tailed tests.

### 2.4. Informed Consent

Written informed consent was obtained from each patient. The study protocol conformed to the ethical guidelines of the 1975 Declaration of Helsinki and was approved by the local institutional review board.

## 3. Results

### 3.1. Baseline Characteristics of CHC Patients

Of 415 CHC patients, 191 (46.0%) were male; 244 (58.8%) and 122 (29.4%) were infected with genotype 1 (G1) and G2 HCV, respectively. In total, 90 (21.6%) had liver cirrhosis and 168 (40.5%) had baseline IR. Compared to patients without baseline IR, those with baseline IR had increased baseline BMI, ALT, and TG levels; HOMA-IR values; prevalence rate of diabetes mellitus; and G1 HCV infection and liver cirrhosis rates but reduced HDL-C levels. (Table 1).

### 3.2. Baseline Associations of CHC Patients: Lipid, Virus, Fibrosis, and IFNL3 Profiles with TC; and TC and BMI with HOMA-IR

At baseline, among the 415 CHC patients, levels of HDL-C, TG, HCV RNA, and IFNL3-rs12979860 CC genotype were positively associated with TC levels, while FIB-4 scores were negatively associated with TC levels. TG and BMI were positively associated with HOMA-IR values (Table 2).

**Figure 2:**
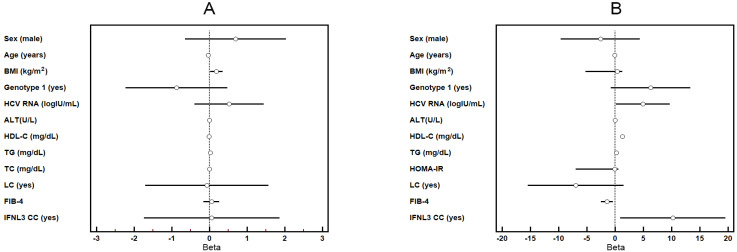
Forrest plot of the Beta and 95% confidence intervals of Beta for HOMA-IR (**A**) and TC (**B**). BMI: body mass index; ALT: alanine aminotransferase; HDL-C: high-density lipoprotein cholesterol; TGs: triglycerides; TC: total cholesterol; LC: liver cirrhosis; FIB-4: fibrosis-4 index; IFNL3 CC: Interferon λ3 CC genotype.

The accompanying Forrest plot for Table 2 was shown in Figure 2.

### 3.3. Posttherapy Associations of SVR Patients: Lipid Profile Consistent with TC and TC Negatively with HOMA-IR

Among the 365 CHC patients who had received and finished a course of DAA therapy, 360 achieved an SVR (98.6%). The post-therapy associations of the SVR patients were as follows.

At 12 weeks after therapy, HDL-C (95% confidence interval of Beta: 0.664~1.361; OR:1.013) and TG (0.165~0.321 (0.243)) levels were positively associated with TC levels, while FIB-4 (−3.387~−0.025 (−1.819)) and HOMA-IR (−1.432~−0.03 (−0.731)) values were negatively associated with TC levels. BMI (0.019~0.382 (0.200)) and TG (0.015~0.044 (0.03)) were positively associated with HOMA-IR, while TC (−0.045~−0.001 (−0.023)) levels were negatively associated with HOMA-IR (Table S2).

At 24 weeks after therapy, HDL-C (0.766~1.515 (1.14)) and TG (0.053~0.163 (0.108)) levels were positively associated with TC levels, while FIB-4 (−4.565~−0.921 (−2.743)) and HOMA-IR (−3.25~−0.303 (−1.777)) values were negatively associated with TC levels. BMI (0.076~0.328 (0.202)), TG (0.000~0.012 (0.006)), and ALT (0.019~0.018 (0.005)) levels were positively associated with HOMA-IR, while TC (−0.034~−0.003 (−0.018)) levels were negatively associated with HOMA-IR (Table S3).

At 48 weeks after therapy, HDL-C (0.554~2.558 (1.556)) and TG (0.059~0.456 (0.258)) levels were positively associated with TC levels. No investigated factors were significantly associated with HOMA-IR at that time point (Table S4).

### 3.4. Alterations of Various Profiles in SVR Patients: Progressively Increased BMI and TG/HDL-C; HOMA-IR Reached Its Nadir at 72 Weeks Post-Therapy

**Figure 3:**
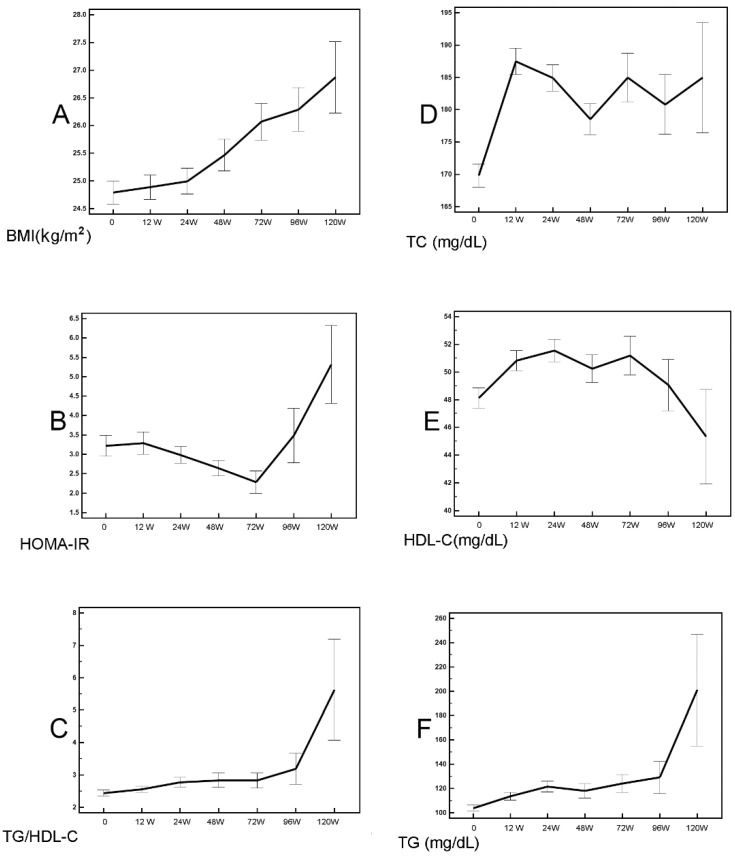
Longitudinal metabolic alteration of SVR patients. Mean ± standard error of body mass index (BMI) (**A**), homeostasis model assessment of insulin resistance (HOMA-IR) (**B**), triglycerides (TGs)/ high-density lipoprotein cholesterol (HDL-C) ratio (**C**), total cholesterol (**D**), HDL-C (**E**), and TGs (**F**). Time point: 0 represents baseline; W represents weeks after completion of anti-HCV therapy. The associated *p* values were showed in Table S5.

During a 3-year follow-up (median: 673 days; mean ± standard deviation: 693.43 ± 159.7 days; range: 413–1095 days), compared with baseline levels, the longitudinal alterations of various variables in SVR patients were as follows: BMI increased significantly beginning 24 weeks after therapy (Figure 3A, Table 3). ALT levels and FIB-4 scores decreased significantly at 12 weeks after therapy and remained stable afterward (Table 3). HOMA-IR trended downward by 72 weeks after therapy (Figure 3B, Table 3) and then increased. TG/HDL-C ratios significantly increased beginning 24 weeks after therapy (Figure 3C, Table 3). TC levels increased and reached a peak at 12 weeks after therapy (*p* < 0.001), with some fluctuations afterward, and the 120-week post-therapy levels were still higher than baseline levels (Figure 3D, Table 3). HDL-C levels increased significantly beginning 12 weeks after therapy, with some fluctuations afterward, and decreased beginning 72 weeks after therapy. The 120-week post-therapy levels were lower than the baseline levels (Figure 3E, Table 3). TG levels increased significantly beginning 12 weeks after therapy (Figure 3F, Table 3).

### 3.5. Various Metabolic Profile Alteration in SVR Patients with and without Baseline IR: Increased BMI but Decreased HOMA-IR Were Noted Earlier in Patients with Than Patients without IR

**Figure 4:**
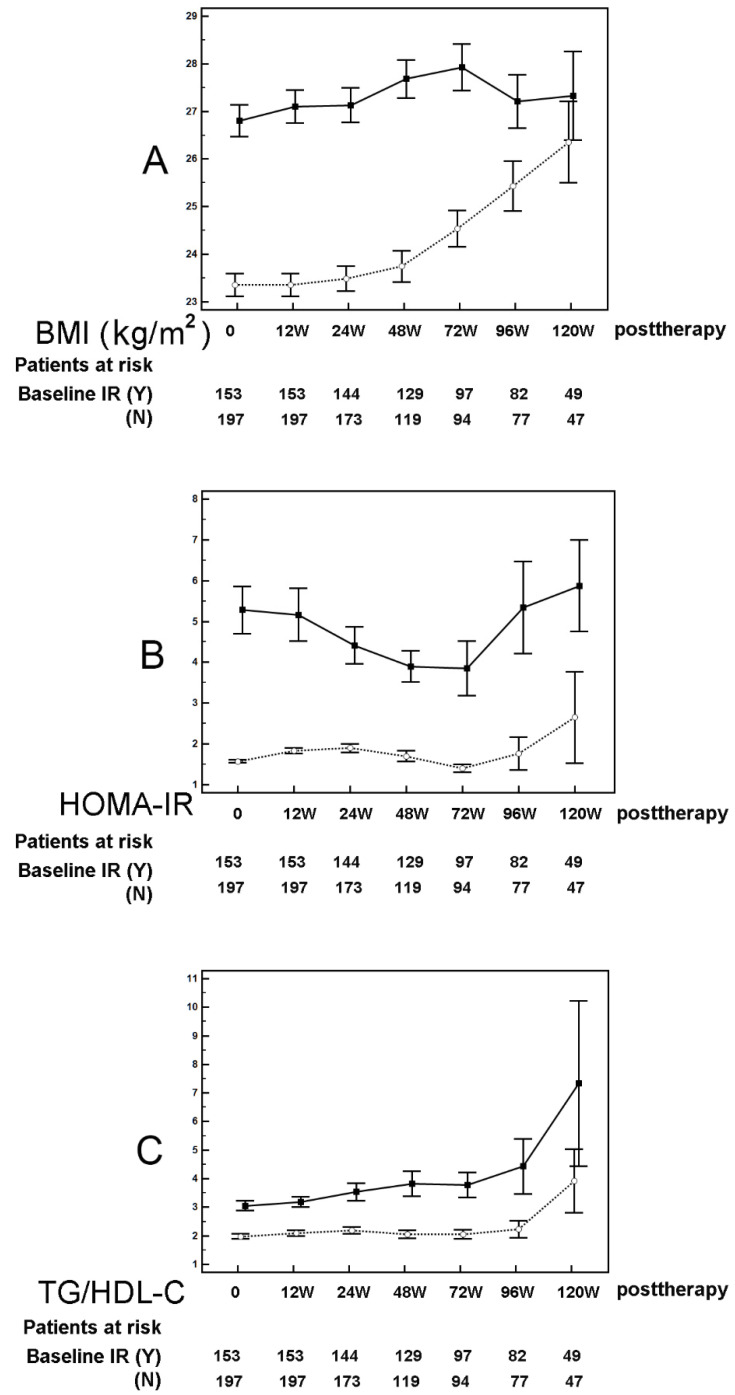
Longitudinal metabolic alteration of SVR patients stratified by baseline insulin resistance (IR). Mean ± standard error of BMI (**A**), HOMA-IR (**B**), and TG/HDL-C ratio (**C**). Time point: 0 represents baseline; W represents weeks after completion of anti-HCV therapy. Baseline IR (Y): SVR patients with baseline IR (solid lines); Baseline IR (N): SVR patients without baseline IR (dashed lines). The associated *p* values are shown in Table S5.

The data from subgroup analyses of SVR patients according to their baseline IR are shown in Figure 4 and Table S5. SVR patients with baseline IR had an increasing trend in BMI from 12 weeks to 72 weeks after therapy, after which their BMI remained steady, while SVR patients without baseline IR did not have an increasing trend until 48 weeks after therapy (Figure 4A, Table S5). For TC levels, the alteration patterns were similar between SVR patients with and without baseline IR: the levels peaked at 12 weeks after therapy, and with some fluctuations afterward (Table S5). Regarding HOMA-IR, SVR patients with baseline IR had a decreasing trend from 12 weeks to 72 weeks after therapy. Beyond this period, HOMA-IR increased. By contrast, among SVR patients without baseline IR, HOMA-IR trended upward from 12 to 24 weeks after therapy, then downward from 48 to 72 weeks after therapy, and finally upward again (Figure 4B, Table S5). SVR patients with and without baseline IR reached their lowest HOMA-IR at 72 weeks after therapy. Regarding TG/HDL-C ratios, SVR patients with and without baseline IR had upward trends, and the increases accelerated beginning 72 weeks after therapy. A more substantial increase was noted in SVR patients with baseline IR than in those without (Figure 4C, Table S5).

### 3.6. Comparisons between Post-Therapy Metabolic Profiles of SVR Patients and Controls: SVR Patients Had Higher BMI and HOMA-IR Levels in the Subgroup without Baseline IR

The baseline and post-therapy profiles of SVR patients were compared with those of sex- and age-matched controls (Figure 1 and Table S6). Of the 470 controls, 216 (46%) were males, 40 (8.5%) had IR, and 22 (4.7%) had diabetes mellitus. Compared with controls, the SVR patients had increased BMI and HOMA-IR values from baseline to 120 weeks after therapy; increased TG levels and TG/HDL-C ratios as well as reduced HDL-C levels from 96 to 120 weeks after therapy; and increased ALT but reduced TC levels at baseline. The differences in TC levels between the controls and SVR patients mostly vanished at 12 weeks after therapy, while the differences in ALT vanished at 12 weeks after therapy, and SVR patients were noted to have higher ALT levels than controls from 24 to 48 weeks after therapy.

The subgroup analyses showed that, within the subgroup with baseline IR, most profiles were similar between the SVR patients and the controls. Within the subgroup without baseline IR, however, the SVR patients had higher HOMA-IR from baseline to 120 weeks after therapy and higher BMI from 96 to 120 weeks after therapy than the controls (Table S6).

## 4. Discussion

Among CHC patients, the baseline positive association between HCV RNA and TC levels coincides with the time when HCV hijacks the cholesterol synthesis pathway to accomplish its life cycle [3], and the negative association between FIB-4 scores and TC levels reflects the fact that severe hepatic fibrosis leads to hypocholesteremia [32], as the liver is the main organ for cholesterol biosynthesis. A link has been shown between the IFNL3-rs12979860 CC genotype and high TC levels [33], while the positive associations of BMI and TG levels with HOMA-IR indicated the utility of BMI and TG in identifying insulin action [34]. Moreover, the increased rates of cirrhosis and G1 HCV in CHC patients with baseline IR were consistent with the facts that IR is associated with significant hepatic fibrosis [35] and that G1 HCV is associated with increased IR [36]. Thus, the baseline associations and comparisons of CHC patients strengthen the reliability of our results.

Increased TC levels [13,14] and BMI [37] have been consistently reported in CHC patients after viral clearance following DAA therapy. By contrast, there is conflicting evidence on whether insulin sensitivity is improved [13,20,21,22,23,24] and how TG and HDL-C levels are altered after SVR following DAA in CHC patients [13,14,15,16,17,18,19]. In the current study, in which a total of 360 SVR patients were followed for years, an initial decreasing trend of HOMA-IR (a proxy of IR if > 2.5) was clearly demonstrated, although it did not become evident until 72 weeks after therapy. Moreover, TG levels were shown to increase with time, and HDL-C levels increased in the beginning but decreased beginning 72 weeks after therapy. An elevated BMI is the core component of metabolic syndrome. Many features of metabolic syndrome are associated with IR [38], and BMI levels are usually positively correlated with HOMA-IR levels [39]. However, we noted increased BMI values but decreased HOMA-IR values among SVR patients by 72 weeks after therapy. As insulin is the chief anabolic hormone, promoting carbon energy deposition in the body [40], the increase in BMI might be a consequence of improved insulin sensitivity, becoming evident earlier than the decrease in HOMA-IR (i.e., decreasing IR). Given that TC levels increased and ALT levels decreased after viral clearance, the negative association of TC levels with HOMA-IR and the positive association of ALT levels with HOMA-IR among SVR patients might account for the initial decreasing trend of HOMA-IR. Increased TC and decreased ALT levels signified improved hepatic cholesterol synthetic capacity and hepatic inflammation, respectively. Namely, improved hepatic function following SVR resulted in improved IR. On the other hand, TC levels usually positively correlate with HOMA-IR [41,42]. However, among SVR patients, TC levels and HOMA-IR were negatively associated with each other. Moreover, although the TG/HDL-C ratio is considered an indicator of IR [30,31], we noted that, in contrast with the decreasing trend in HOMA-IR by 72 weeks after therapy, TG/HDL-C increased with time among the SVR patients. These intriguing facts suggest that the processes of lipid and glucose metabolism were decoupled during the recovery from HCV-induced metabolic alterations, at least by 72 weeks after therapy [3]. Specifically, when we stratified the SVR patients by baseline IR, 12 weeks after therapy was the first critical point when TC levels peaked, and patients with and without baseline IR had decreased and increased HOMA-IR, respectively. Moreover, an earlier increase in BMI and a more substantial increase in TG/HDL-C ratios were noted in SVR patients with than in those without baseline IR. Additionally, compared with controls, increased levels of HOMA-IR and BMI of SVR patients were evident only in the subgroup without baseline IR. Thus, SVR patients with baseline IR benefited from improved IR and recovered from HCV-associated metabolic alterations earlier than SVR patients without baseline IR. For all SVR patients, regardless of baseline IR, 72 weeks after therapy was a second critical point, when HOMA-IR reached its nadir. Beginning 72 weeks after therapy, HDL-C levels tended to decrease, and BMI, HOMA-IR, and TG/HDL-C ratios all increased with time. All of the above results suggested that the initially decoupled processes of lipid and glucose metabolism were re-coupled beginning 72 weeks after therapy. Although the improvement in liver stiffness in SVR patients, evident by decreased FIB-4 scores compared with baseline, was consistent with a previous study [30], there was a tendency for the controlled attenuation parameter, an indicator of hepatic steatosis [31], to increase from baseline in response to DAA therapy in SVR patients [30]. This observation was compatible with the fact that BMI and TG constantly trended upward in SVR patients, who were noted to have higher ALT levels than controls during the latter part of the follow-up period. Increased BMI and TG levels and hepatic steatosis [31] might ultimately dampen the beneficial effects of improved IR among SVR patients, and the trend of decreased HOMA-IR finally reversed 72 weeks after therapy. Collectively, the different paces of metabolic recovery between SVR patients with and without baseline IR, in addition to the long process of decoupling and re-coupling of glucose and lipid metabolism, might account for the discrepancies among studies regarding the HOMA-IR [18,25,30,31,32,33] and lipid level [18,19,20,21,22,23,24] alterations in SVR patients because most studies did not stratify the patients with baseline IR and followed the patients for only 12-48 weeks after therapy.

Taken together, in SVR patients following DAA therapy, the recovered lipid and glucose metabolism was not coupled until 72 weeks after therapy, when the HOMA-IR index reached its nadir. The SVR patients with baseline IR recovered earlier than those without baseline IR. These characteristic metabolic recovery patterns might merit further study as targets for preventing or treating cardiometabolic events and diabetes in SVR patients.

## Figures and Tables

**Table 1:** Baseline characteristics of the CHC patients.

	Total (*n* = 415)	Baseline IR (+) (*n* = 168)	Baseline IR (−) (*n* = 247)	*p* Values
Sex (Male), *n* (%)	191 (46)	85 (50.6)	102 (43.6)	0.099
Age (years)	60.33 ±12.83	59.14 ± 12.95	61.38 ± 12.64	0.085
BMI (kg/m^2^)	24.69 ±34.92	26.72 ± 4.04	23.29 ± 3.18	<0.001
HCV genotype 1, *n* (%)	244 (58.8)	107 (63.7)	131 (56.0)	0.031
HCV genotype 2, *n* (%)	122 (29.4)	42 (25.0)	76 (32.5)	0.088
Log HCV RNA (logIU/mL)	5.98 ± 0.87	6.01 ± 0.87	5.97 ± 0.88	0.612
ALT(U/L)	80.93 ± 91.26	93.83 ± 118.9	73.12 ± 65.99	0.027
HOMA-IR	3.21 ± 4.96	5.47 ± 7.07	1.59 ± 0.54	<0.001
HDL-C (mg/dL)	48.27 ± 13.82	44.02 ± 10.97	51.10 ±14.67	<0.001
TG (mg/dL)	102.26 ± 52.47	120.1 ± 63.3	89.57 ± 38.81	<0.001
TC (mg/dL)	169.46 ± 34.91	169.7 ± 33.92	168.44 ± 34.38	0.716
Liver cirrhosis, *n* (%)	90 (21.6)	48 (28.57)	42 (17)	0.007
Diabetes mellitus, *n* (%)	52 (12.5)	48 (28.5)	4 (1.6)	0.004
FIB-4	3.52 ±3.39	3.56 ± 3.44	3.58 ± 3.42	0.955
IFNL3-rs12979860 CC genotype, *n* (%)	351 (84.6)	141 (83.3)	210 (85)	0.527

CHC: chronic hepatitis C virus infection; IR: insulin resistance; BMI: body mass index; RNA: ribonucleic acid; ALT: alanine transaminase; HOMA-IR: homeostatic model assessment insulin resistance; HDL-C: high-density lipoprotein-cholesterol; TGs: triglycerides; TC: total cholesterol; FIB-4: Fibrosis-4 index; IFNL3: interferon-λ3.

**Table 2:** Multivariate analyses for factors of HOMA-IR and TC levels in CHC patients at baseline.

	HOMA-IR		TC (mg/dL)	
	Beta (95% CI of Beta)	*p* Values	Beta (95% CI of Beta)	*p* Values
Sex (male)	0.693 (−0.643~2.029)	0.308	−2.612 (−9.588~4.364)	0.462
Age (years)	−0.033 (−0.087~0.021)	0.225	−0.039 (−0.32 ~0.242)	0.787
BMI (kg/m2)	0.187 (0.018~0.357)	0.03	0.371 (−5.19~1.261)	0.412
Genotype 1 (yes)	−0.879 (−2.23~0.474)	0.202	6.293 (−0.749~13.334)	0.08
Log HCV RNA (logIU/mL)	0.521 (0.397~1.439)	0.265	4.944 (0.182~9.706)	0.042
ALT(U/L)	−0.001 (−0.007~0.006)	0.869	0.012 (−0.022~0.047)	0.478
HDL-C (mg/dL)	−0.008 (−0.07~0.054)	0.807	1.282 (1.000~1.564)	<0.001
TG (mg/dL)	0.019 (0.004~0.033)	0.011	0.222 (0.15~0.294)	<0.001
TC (mg/dL)	−0.002 (−0.016~0.013)	0.851	NA	
HOMA-IR	NA		−0.061 (−6.99~0.577)	0.851
Liver cirrhosis (yes)	−0.067 (−1.699~1.564)	0.935	−6.967 (−15.433~1.500)	0.106
FIB-4	0.054 (−0.155~0.262)	0.612	−1.459 (−2.53~−0.386)	0.008
IFNL3 CC genotype (yes)	0.06 (−1.744~1.863)	0.948	10.24 (0.919~19.565)	0.031

CHC: chronic hepatitis C virus infection; CI: confidence interval; OR: odds ratio; BMI: body mass index; RNA: ribonucleic acid; ALT: alanine transaminase; HOMA-IR: homeostatic model assessment insulin resistance; HDL-C: high-density lipoprotein-cholesterol; TGs: triglycerides; TC: total cholesterol; NA: not assessable; FIB-4: fibrosis-4 index; IFNL3: interferon-λ3.

**Table 3:** Comparisons of various profile values between different time points of the same SVR patients.

	12-Week Posttherapy vs. Baseline (*n* = 360)		24-Week Posttherapy vs. Baseline (*n* = 317)		48-Week Posttherapy vs. Baseline (*n* = 248)		72-Week Posttherapy vs. Baseline (*n* = 191)		96-Week Posttherapy vs. Baseline (*n* = 159)		120-Week Posttherapy vs. Baseline (*n* = 96)	
	Difference	*p* Values	Difference	*p* Values	Difference	*p* Values	Difference	*p* Values	Difference	*p* Values	Difference	*p* Values
BMI (kg/m^2^)	0.09 ± 0.06	0.111	0.2 ± 0.07	0.007	0.48 ± 0.10	<0.001	0.76 ± 0.12	<0.001	0.97 ± 0.183	<0.001	1.57 ± 0.292	<0.001
ALT(U/L)	−58.9 ± 4.62	<0.001	−60.2 ± 4.94	<0.001	−66.6 ± 6.44	<0.001	−59.8 ± 5.00	<0.001	−57.7 ± 7.18	<0.001	−63.7 ± 10.6	<0.001
FIB-4	−0.71 ± 0.08	<0.001	−0.32 ± 0.15	<0.001	−1.25 ± 0.214	<0.001	−1.12 ± 0.40	<0.001	−1.82 ± 0.37	<0.001	−1.05 ± 3.14	0.007
TC (mg/dL)	17.6 ± 1.44	<0.001	14.4 ± 1.60	<0.001	10.3 ± 1.84	<0.001	14.5 ± 3.05	<0.001	13.4 ± 3.72	0.001	22.3 ± 8.63	0.018
TG (mg/dL)	9.82 ± 2.58	<0.001	18.7 ± 3.63	<0.001	19.4 ± 5.11	<0.001	25.6 ± 5.61	<0.001	29.6 ± 8.45	<0.001	38.9 ± 9.6	<0.001
HDL-C (mg/dL)	2.86 ± 0.48	<0.001	3.09 ± 5.21	<0.001	1.80 ± 0.64	0.005	2.89 ± 0.92	0.002	2.13 ± 0.97	0.004	−0.48 ± 2.01	0.034
TG/HDL-C	0.10 ± 0.07	0.191	0.38 ± 0.11	0.001	0.55 ± 0.18	0.003	0.53 ± 0.16	0.001	0.77 ± 0.29	0.011	2.76 ± 1.10	0.021
HOMA-IR	0.11 ± 0.23	0.626	−0.22 ± 0.31	0.479	−0.77 ± 0.52	0.147	−0.41 ± 0.19	0.039	0.38 ± 0.14	0.436	1.03 ± 0.68	0.16

BMI: body mass index; ALT: alanine transaminase; HOMA-IR: homeostatic model assessment insulin resistance; HDL-C: high-density lipoprotein-cholesterol; TGs: triglycerides; TC: total cholesterol; FIB-4: fibrosis-4 index.

## Data Availability

Data available on reasonable request from the authors.

## Supplementary Materials

The following are available online at www.mdpi.com/article/10.3390/cells10112934/s1, Table S1:Various DAA combinations used in the study.; Table S2: Multivariate analyses for factors of HOMA-IR and TC levels in SVR patients at 12 weeks posttherapy; Table S3: Multivariate analyses for factors of HOMA-IR and TC levels in SVR patients at 24 weeks posttherapy; Table S4: Multivariate analyses for factors of HOMA-IR and TC levels in SVR patients at 48 weeks posttherapy; Table S5: Comparisons of various profiles between different time points of the same SVR patients with and without IR; Table S6: Comparisons of various profiles between the SVR and sex-, and age-matched control subjects.
