# Supplementary material for: Decoupled Glucose and Lipid Metabolic Recovery after Viral Clearance in Direct-Acting Antiviral-Treated HCV Patients: A 3-Year Prospective Cohort Study
Source: Cells. 2021 Oct 28;10(11):2934. doi: 10.3390/cells10112934 (PMC8616092; doi:10.3390/cells10112934)
Supplement: Supplementary file 1 [file cells-10-02934-s001.zip › cells-1442069-supplementary.pdf]

Table S1. Various DAA combinations used in the study.

Genotype 1		Genotype 2		Genotype 3		Genotype 4		Genotype 5		Genotype 6	
DAA	D(W)	DAA	D(W)	DAA	D(W)	DAA	D(W)	DAA	D(W)	DAA	D(W)
Asunaprevir (100 mg, bid) with Daclatasvir (60 mg, qd)	24	Sovaldi (Sofosbuvir 400 mg qd) with Ribavirin	12								
Viekirax (Ombitasvir 12.5mg/Paritaprevir 75mg/Ritonavir 50 mg qd) with Exviera (250 mg bid)	12-24										
Zepatier (Elbasvir 50 mg/Grazoprevir 100 mg, qd)	12-16										
Harvoni (Ledipasvir 90 mg /Sofosbuvir 400 mg qd) with/without Ribavinin (800-1400 mg/day, in two divided doses	12	Harvoni (Ledipasvir 90 mg /Sofosbuvir 400 mg qd) with/without Ribavinin (800-1400 mg/day, in two divided doses	12			Harvoni (Ledipasvir 90 mg /Sofosbuvir 400 mg qd) with/without Ribavinin (800-1400 mg/day, in two divided	12	Harvoni (Ledipasvir 90 mg /Sofosbuvir 400 mg qd) with/without Ribavinin (800-1400 mg/day, in two divided	12	Harvoni (Ledipasvir 90 mg /Sofosbuvir 400 mg qd) with/without Ribavinin (800-1400 mg/day, in two divided	12
Mavyret	8-16	Mavyret	8-12	Mavyret	8-16	Mavyret	8-12	Mavyret	8-12	Mavyret	8-12

(glecaprevir 100mg/pibrentasvir 40mg, 3# qd)		(glecaprevir 100mg/pibrentasvir 40mg, 3# qd)		(glecaprevir 100mg/pibrentasvir 40mg, 3# qd)		(glecaprevir 100mg/pibrentasvir 40mg, 3# qd)		(glecaprevir 100mg/pibrentasvir 40mg, 3# qd)		(glecaprevir 100mg/pibrentasvir 40mg, 3# qd)	
Epclusa (sofosbuvir 400 mg/velpatasvir 100 mg qd) with or without Ribavirin	12	Epclusa (sofosbuvir 400 mg/velpatasvir 100 mg qd) with or without Ribavirin	12	Epclusa (sofosbuvir 400 mg/velpatasvir 100 mg qd) with or without Ribavirin	12	Epclusa (sofosbuvir 400 mg/velpatasvir 100 mg qd) with or without Ribavirin	12	Epclusa (sofosbuvir 400 mg/velpatasvir 100 mg qd) with or without Ribavirin	12	Epclusa (sofosbuvir 400 mg/velpatasvir 100 mg qd) with or without Ribavirin	12

DAA: direct-acting antiviral; D: duration: W: weeks.

Table S2. Multivariate analyses for factors of HOMA-IR and TC levels in SVR patients at 12 weeks posttherapy.

	HOMA-IR		TC (mg/dL)	
	95% CI of Beta (Beta)	*p* values	95% CI of Beta (Beta)	*p* values
Sex (male)	-2.03~0.891 (-0.57)	0.443	-13.018~3.366 (-4.826)	0.247
Age (years)	-0.068 ~0.04 (-0.014)	0.617	-0.300 ~0.308 (0.004)	0.979
BMI (kg/m^2)	0.019~0.382 (0.200)	0.031	-0.979~1.078 (0.049)	0.925
ALT(U/L)	-0.012~0.076 (0.032)	0.149	-0.177~0.314 (0.068)	0.584
HDL-C (mg/dL)	-0.025 ~0.107 (0.041)	0.219	0.664 ~1.361 (1.013)	< 0.001
TG (mg/dL)	0.015~0.044 (0.03)	<0.001	0.165 ~0.321 (0.243)	< 0.001
TC (mg/dL)	-0.045 ~-0.001 (-0.023)	0.041	NA	
HOMA-IR	NA		-1.432~-0.03 (-0.731)	0.041
Liver cirrhosis (yes)	-1.023~2.392 (0.685)	0.43	-18.8~0.231 (-9.299)	0.056
FIB-4	-0.233 ~0.331 (0.049)	0.733	-3.387 ~-0.025 (-1.819)	0.023
IFNL3 CC genotype (yes)	-0.762~2.907 (1.073)	0.251	-12.827~8.633 (-1.697)	0.747

SVR: sustained virological response; CI: confidence interval; BMI: body mass index; ALT: alanine transaminase; HOMA-IR: homeostatic model assessment insulin resistance; HDL-C: high density lipoprotein-cholesterol; TG: triglycerides; TC: total cholesterol; NA: not assessable; FIB-4: Fibrosis-4 index; IFNL3: interferon-λ3.

Table S3. Multivariate analyses for factors of HOMA-IR and TC levels in SVR patients at 24 weeks posttherapy.

	HOMA-IR		TC (mg/dL)	
	95% CI of Beta (Beta)	*p* values	95% CI of Beta (Beta)	*p* values
Sex (male)	-0.953~1.092 (0.069)	0.894	-15.71~7.26 (-5.73)	0.259
Age (years)	-0.078 ~0.001 (-0.039)	0.056	-0.55 ~0.236 (-0.157)	0.431
BMI (kg/m^2)	0.076 ~0.328 (0.202)	0.002	-0.575~1.957 (0.691)	0.283
ALT(U/L)	0.019~0.018 (0.005)	0.001	-0.198 ~0.412 (0.107)	0.49
HDL-C (mg/dL)	-0.021 ~0.063 (0.021)	0.316	0.766 ~1.515 (1.14)	<0.001
TG (mg/dL)	0.000~0.012 (0.006)	0.037	0.053~0.163 (0.108)	<0.001
TC (mg/dL)	-0.034~-0.003 (-0.018)	0.018	NA	
HOMA-IR	NA		-3.25~-0.303 (-1.777)	0.018
Liver cirrhosis (yes)	-0.844~1.406 (0.281)	0.623	-19.54~2.379 (-8.579)	0.124
FIB-4	-0.115~0.265 (0.075)	0.438	-4.565~-0.921 (-2.743)	0.003
IFNL3 CC genotype (yes)	-0.454~0.173 (0.86)	0.198	-9.013~16.851 (3.919)	0.916

SVR: sustained virological response; CI: confidence interval; BMI: body mass index; ALT: alanine transaminase; HOMA-IR: homeostatic model assessment insulin resistance; HDL-C: high density lipoprotein-cholesterol; TG: triglycerides; TC: total cholesterol; NA: not assessable; FIB-4: Fibrosis-4 index; IFNL3: interferon-λ3.

Table S4. Multivariate analyses for factors of HOMA-IR and TC levels in SVR patients at 48 weeks posttherapy.

	HOMA-IR		TC (mg/dL)	
	95% CI of Beta (Beta)	*p* values	95% CI of Beta (Beta)	*p* values
Sex (male)	-11.368~3.698 (-3.836)	0.248	-28.89~34.44 (4.269)	0.758
Age (years)	-0.313 ~0.141 (-0.086)	0.375	-0.215 ~1.773 (0.779)	0.119
BMI (kg/m^2)	0.608 ~1.813 (0.602)	0.257	-4.691~2.59 (-1.05)	0.558
ALT(U/L)	-0.178 ~0.319 (0.070)	0.499	-1.078~0.577 (-0.251)	0.539
HDL-C (mg/dL)	-0.168 ~0.02 (-0.074)	0.523	0.554 ~2.558 (1.556)	0.004
TG (mg/dL)	-0.016~0.021 (0.003)	0.228	0.059~0.456 (0.258)	0.013
TC (mg/dL)	-0.022 ~0.044 (0.011)	0.924	NA	
HOMA-IR	NA		-3.178~6.422 (1.622)	0.493
Liver cirrhosis (yes)	-4.452~0.900 (-1.776)	0.514	-48.01~17.41 (-15.29)	0.345
FIB-4	-0.196~1.32 (0.562)	0.677	-14.22~2.378 (-15.296)	0.287
IFNL3 CC genotype (yes)	-2.292~2.659 (0.184)	0.626	-45.72~12.249 (-16.73)	0.246

SVR: sustained virological response; CI: confidence interval; BMI: body mass index; ALT: alanine transaminase; HOMA-IR: homeostatic model assessment insulin resistance; HDL-C: high density lipoprotein-cholesterol; TG: triglycerides; TC: total cholesterol; NA: not assessable; FIB-4: Fibrosis-4 index; IFNL3: interferon-λ3.

Table S5. Comparisons of various profiles between different time points of the same SVR patients with and without IR.

	12-week posttherapy vs. baseline		24-week posttherapy vs. baseline		48-week posttherapy vs. baseline		72-week posttherapy vs. baseline		96-week posttherapy vs. baseline		120-week posttherapy vs. baseline	
	Differences	*p* values	Differences	*p* values	Differences	*p* values	Differences	*p* values	Differences	*p* values	Differences	*p* values
**BMI (kg/m^2)**												
Baseline IR	0.26±1.15	0.005	0.33±1.22	0.002	0.73±1.35	<0.001	0.88±1.67	<0.001	1.01±2.09	0.001	1.70±1.78	0.001
Baseline non-IR	0.18±3.32	0.435	0.11±1.32	0.269	0.27±1.40	0.032	0.66±1.56	<0.001	0.92±1.41	<0.001	1.40±1.56	0.005
**TC (mg/dL)**												
Baseline IR	17.52±28.5	<0.001	13.07±29.26	<0.001	6.78±30.71	0.03	8.69±36.19	0.046	10.54±29.65	0.039	19.09±48.37	0.035
Baseline non-IR	18.69±28.24	<0.001	15.04±28.56	<0.001	13.80±24.12	<0.001	19.41±28.30	<0.001	15.42±27.26	0.003	25.90±29.24	0.021
**HOMA-IR**												
Baseline IR	-0.161±0.31	0.003	-0.92±0.79	<0.001	-1.76±9.39	<0.001	-0.82±2.02	0.043	0.49±2.92	0.859	1.39+/-0.075	0.432
Baseline non-IR	0.252±0.85	0.001	0.31±1.41	0.02	0.09±1.09	0.531	-0.18±0.67	0.049	0.27±1.02	0.919	1.010±0.65	0.18
**TG/HDL-C**												
Baseline IR	0.134±1.93	0.395	0.565±2.83	0.020	0.98±3.69	0.011	0.891±2.033	0.003	1.162±2.799	0.027	3.49±6.45	0.011
Baseline non-IR	0.080±1.078	0.298	0.214±1.23	0.021	0.200±1.31	0.101	0.239±1.22	0.012	0.440±1.044	0.037	2.025±2.88	0.044

BMI: body mass index; IR: insulin resistance; TC: total cholesterol; HOMA-IR: homeostatic model assessment insulin resistance; TG: triglycerides; HDL-C: High-density lipoprotein-cholesterol.

Table S6. Comparisons of various profiles between the SVR and sex-, and age-matched control subjects

	Control	Baseline	*p* values	12W SVR	*p* values	24W SVR	*p* values	48W SVR	*p* values	72W SVR	*p* values	96W SVR	*p* values	120W SVR	*p* values
**BMI (kg/m²)**	23.55±2.97	24.71±3.98	<0.001	24.82±4.17	<0.001	24.90±4.16	<0.001	25.04±4.29	<0.001	25.54±4.34	<0.001	25.75±3.19	<0.001	25.50±2.98	<0.001
Baseline IR	26.50±2.67	26.69±4.16	0.854	27.07±4.36	0.47	27.03±4.27	0.623	27.59±3.98	0.289	27.60±4.56	0.35	26.48±3.34	0.986	25.73±3.15	0.486
Baseline non-IR	23.28±2.78	23.29±3.09	0.986	23.22±3.23	0.855	23.38±3.37	0.777	23.05±3.39	0.560	23.81±3.30	0.21	24.90±2.84	0.005	25.10±2.84	0.002
**ALT(U/L)**	21.28±12.0	83.4±101.5	<0.001	23.3±14.93	0.081	24.1±16.69	0.027	25.12±18.1	0.017	31.2±37.04	0.003	29.9±43.37	0.136	25.0±12.32	0.183
Baseline IR	33.17±25.4	96.39±135.	<0.001	26.69±16.4	0.17	27.89±17.5	0.29	32.79±21.8	0.949	37.91±32.5	0.583	42.11±60.1	0.565	27.76±13.8	0.496
Baseline non-IR	20.54±10.2	75.8±68.98	<0.001	21.0±13.38	0.691	21.6±15.81	0.450	18.94±11.5	0.241	25.77±39.7	0.281	18.63±7.49	0.328	19.87±7.19	0.861
**HDL-C (mg/dL)**	53.1±13.08	48.7±14.56	<0.001	51.9±14.24	0.301	52.95±15.1	0.875	50.6±14.81	0.074	52.6±14.99	0.764	46.90±9.72	0.012	41.58±9.88	0.003
Baseline IR	50.00±15.7	43.44±10.7	0.029	46.56±11.8	0.295	47.31±11.9	0.42	45.23±13.4	0.25	44.47±10.4	0.142	43.78±9.85	0.210	39.57±6.18	0.107
Baseline non-IR	53.55±13.2	52.41±15.4	0.445	55.37±14.2	0.23	56.82±16.1	0.058	54.42±14.3	0.624	58.67±15.0	0.022	49.62±9.04	0.245	44.40±13.9	0.129
**TG (mg/dL)**	109.8±70.9	106.8±63.2	0.596	114.8±60.8	0.393	121.2±86.4	0.104	118.7±97.0	0.324	114.5±69.9	0.598	141.4±115.	0.028	233.4±252	<0.001
Baseline IR	141.2±54.9	124.6±69.0	0.342	132.9±65.7	0.623	143.4±111.	0.939	147.4±128.	0.847	133.6±88.6	0.748	189.3±157.	0.249	309.4±314.	0.039
Baseline non-IR	107.3±75.8	92.6±51.7	0.034	100.8±53.2	0.388	104.3±55.1	0.703	97.3755.77	0.281	100.4±48.4	0.556	104.2±44.0	0.863	127.0±44.7	0.565
**TC (mg/dL)**	188.5±33.3	174.5±37.4	<0.001	192.0±40.4	0.281	189.5±37.9	0.757	180.6±36.4	0.025	186.7±42.8	0.729	180.4±37.3	0.195	187.3±32.4	0.894
Baseline IR	192.5±42.5	171.4±36.0	0.29	188.5±37.5	0.693	183.8±32.2	0.334	178.6±38.0	0.195	176.9±40.2	0.206	180.3±36.0	0.384	191.2±31.0	0.94
Baseline non-IR	188.0±32.5	175.0±374	<0.001	192.6±39.0	0.266	191.9±38.6	0.346	181.9±35.3	0.156	194.2±43.6	0.379	180.5±39.7	0.371	181.0±37.3	0.632
**HOMA-IR**	1.39±1.044	3.34±5.56	<0.001	3.16±4.96	<0.001	2.89±3.25	<0.001	2.47±2.35	<0.001	2.47±2.23	0.009	3.54±2.54	0.01	3.46±2.54	0.049
Baseline IR	4.09±2.0	5.70±7.9	0.421	4.93±7.21	0.624	4.20±4.33	0.92	3.93±3.03	0.852	4.60±2.85	0.584	4.86±2.37	0.409	5.46±2.855	0.765
Baseline non-IR	1.18±0.51	1.59±0.53	<0.001	1.87±0.92	<0.001	1.99±1.627	<0.001	1.60±1.176	0.008	1.450±.612	0.02	1.49± 0.67	0.03	2.25±3.42	0.025
**TG/HDL-C**	2.33±2.19	2.55±2.072	0.232	2.51±1.80	0.311	2.68±2.75	0.111	2.86±3.58	0.105	2.49±2.12	0.562	3.62±3.84	0.006	6.53±8.35	<0.001
Baseline IR	3.11±1.50	3.17±2.25	0.904	3.19±2.07	0.872	3.49±3.71	0.678	3.95±4.99	0.495	3.33±2.80	0.756	5.01±5.12	0.155	8.88±10.50	0.033
Baseline non-IR	2.28±2.37	2.06±1.61	0.3	2.02±1.38	0.244	2.09±1.501	0.43	2.06±1.60	0.434	1.87±1.11	0.258	2.40±1.56	0.847	3.25±1.81	0.369

SVR: sustained virological response; BMI: body mass index; ALT: alanine transaminase; HDL-C: high density lipoprotein-cholesterol; TG: triglycerides; TC: total cholesterol; HOMA-IR: homeostatic model assessment insulin resistance. IR: insulin resistance; Control: data from sex- and age-matched controls; W: weeks posttherapy; *p* values: p values of the comparisons between controls and SVR patients.
